# Essences in Metabolic Engineering of Lignan Biosynthesis

**DOI:** 10.3390/metabo5020270

**Published:** 2015-05-04

**Authors:** Honoo Satake, Tomotsugu Koyama, Sedigheh Esmaeilzadeh Bahabadi, Erika Matsumoto, Eiichiro Ono, Jun Murata

**Affiliations:** 1 Bioorganic Research Institute, Suntory Foundation for Life Sciences, Osaka 618-8503, Japan; E-Mails: koyama@sunbor.or.jp (T.K.); matsumoto@sunbor.or.jp (E.M.); murata-j@sunbor.or.jp (J.M.); 2 Department of Biology, Faculty of Basic Sciences, University of Zabol, Zabol, Iran; E-Mail: shirin_esm@yahoo.com; 3 Research Institute, Suntory Global Innovation Center (SIC) Ltd., Osaka 618-8503, Japan; E-Mail: eiichiro_ono@suntory.co.jp

**Keywords:** lignan, biosynthesis, metabolic engineering, elicitor, transgenic plant

## Abstract

Lignans are structurally and functionally diverse phytochemicals biosynthesized in diverse plant species and have received wide attentions as leading compounds of novel drugs for tumor treatment and healthy diets to reduce of the risks of lifestyle-related non-communicable diseases. However, the lineage-specific distribution and the low-amount of production in natural plants, some of which are endangered species, hinder the efficient and stable production of beneficial lignans. Accordingly, the development of new procedures for lignan production is of keen interest. Recent marked advances in the molecular and functional characterization of lignan biosynthetic enzymes and endogenous and exogenous factors for lignan biosynthesis have suggested new methods for the metabolic engineering of lignan biosynthesis cascades leading to the efficient, sustainable, and stable lignan production in plants, including plant cell/organ cultures. Optimization of light conditions, utilization of a wide range of elicitor treatments, and construction of transiently gene-transfected or transgenic lignan-biosynthesizing plants are mainly being attempted. This review will present the basic and latest knowledge regarding metabolic engineering of lignans based on their biosynthetic pathways and biological activities, and the perspectives in lignan production via metabolic engineering.

## 1. Introduction

A tremendous increase in the number of elderly individuals has caused a rapid escalation of medical care expenses. This may eventually lead to a serious disruption in essential medical care systems and national financial burdens. To address these issues, extensive efforts are therefore underway to increase the healthy life expectancy, prevent lifestyle-related diseases, and make progress in medical treatments. Consequently, the consistent and appropriate intake of dietary supplements and the efficient development of clinical drugs are the most promising and effective ways to achieve these goals.

**Figure 1:**
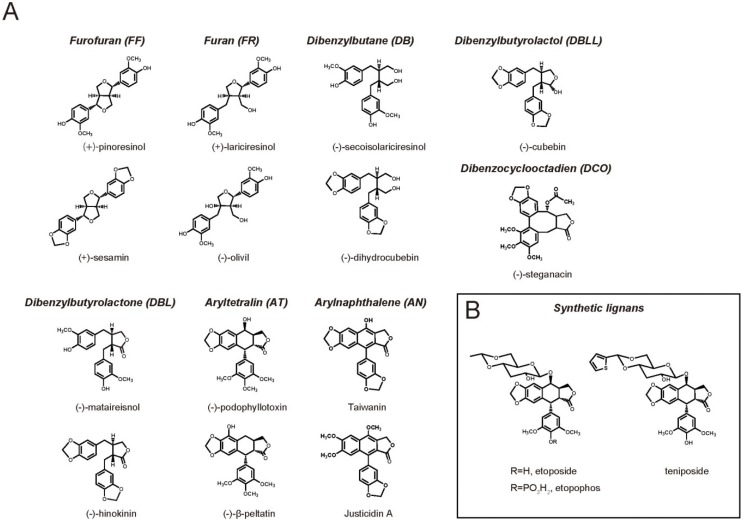
Chemical Structures of Typical Lignans in (**A**) Dietary and Medicinal Sources and (**B**) Synthetic Podophyllotoxin Derivatives.

Dietary supplements and drug compounds are largely derived from specialized metabolites, previously called secondary metabolites of plants, including alkaloids, flavonoids, isoflavonoids, and lignans. As depicted in Figure 1A, lignans are naturally occurring phenylpropanoid dimers (C6-C3 unit; e.g., coniferyl alcohol), in which the phenylpropane units are linked by the central carbons of the side chains. These specialized metabolites are classified into eight groups based on their structural patterns, including their carbon skeletons; the way in which oxygen is incorporated into the skeletons; and the cyclization pattern: furofuran, furan, dibenzylbutane, dibenzylbutyrolactone, aryltetralin, arylnaphthalene, dibenzocyclooctadiene, and dibenzylbutyrolactol [1,2]. Unfortunately, the amounts of lignans and their precursor molecules in model plants such as *Arabidopsis thaliana* and *Nicotiana tabacum* are quite low. Moreover, plant sources of lignans are frequently limited because of the high cost of plant hunting and collection, poor cultivation systems, long growth phase, and the low lignan content [1,2,3,4,5,6,7,8,9,10,11,12]. For instance, sesamin, a multifunctional sesame seed lignan, is extracted from sesame seed oil, the most abundant source of this compound. Nevertheless, sesamin at most constitutes 0.4%–0.6% (w/w) of sesame seed oil. Moreover, sesame seeds are cultivated only once per year, limiting the ability to obtain large amounts of this compound. Likewise, podophyllotoxin (PTOX), a lignan that is a precursor of semi-synthetic anti-tumor drugs, is isolated from the roots and rhizomes of *Podophyllum hexandrum*, which is distributed in very limited regions, and is now endangered due to overharvesting and environmental disruption [13]. In addition, the complicated chemical structures of PTOX and the related compounds (Figure 1A) make stereoselective organic synthesis impractical and costly for producing large supplies of these compounds and the resulting high cost [1,2,3,4,5,6,7,8,9,10,11,12]. These drawbacks indicate the requirement for efficient, stable and sustainable production systems for producing lignans.

There has been a growing body of reports on the molecular characterization of the enzymes involved in the biosynthesis of lignans, lignan-production using lignan-rich plants or cultured plant cells, including *Linum*, *Forsythia*, and *Podophyllum* species [14,15,16,17,18], and the physiological analyses of their biological activities in mammals. These findings have allowed us to attempt the metabolic engineering of lignan biosynthesis in *Linum*, *Forsythia*, and *Podophyllum* species. This review article will provide current knowledge of lignan production via metabolic engineering and perspectives in the development of metabolic engineering-based lignan production.

## 2. Lignan Biological Activity on Mammals

Although lignans exhibit a wide variety of bioactivities on plants, insects, and mammals [12,19,20,21,22,23,24], they are of especial interest due to the unique antitumor-associated activities and reduction of lifestyle-related diseases. Lignans and their glycosides, including pinoresiniol, sesamin, lariciresinol, secoisolariciresinol, and matairesinol, are metabolized by intestinal microflora to yield enterodiol and enterolactone, which are well known as enterolignans or mammalian lignans [25,26,27]. These metabolized lignans elicited their modest estrogen-like activity in mammals. For example, enterolignans bind to the mammalian estrogen receptors (ER), ERα or ERβ, which are key regulatory factors in the sexual maturation of genital organs [28,29]. Consequently, enterolignans, combined with other intestinal flora-generating metabolites of isoflavones and coumestans, are also called phytoestrogens.

It should also be noteworthy that low concentration of intact lignans have been detected in the sera of mammals fed with lignan-rich diets, suggesting that non-metabolized lignans are taken up by the mammalian digestive system and manifest ER-independent activities *in vivo* and *in vitro*, including tumor growth suppression, angiogenesis inhibition, and reduction of diabetes [6,30,31,32,33,34,35].

Lignans have also been shown to exhibit positive effects on other lifestyle-related diseases. Administration of flaxseed lignan complexes improved hyperglycemia and markers of type II diabetes in elderly patients and various animal models [36,37]. In particular, secoisolariciresinol diglucosides (SDG), secoisolariciresinol, enterodiol and enterolactone inhibited pancreatic α-amidase activity in a non-competitive manner [38]. Sesamin and its metabolites exhibited anti-hypertensive activities [39,40,41]. The anti-oxidative propensity of sesamin is also likely to be involved in protecting the liver from oxidation by alcohols, lipids, and oxygen radicals [39,42,43,44]. In human intestinal Caco 2 cells, pinoresinol decreased the production of inflammatory factors, such as interleukin-6 and prostaglandin E2, following the down-regulation of Cox-2, an inducible prostaglandin synthase that is responsible for the synthesis of prostaglandin H, a precursor of any other prostaglandins [30]. In contrast, matairesinol increased levels of prostaglandin E2 [30]. These findings proved that pinoresinol and matairesinol have opposite effects in these cells [30].**

Of the most prominent epidemiological significance is that intake of lignan-rich foods, such as flaxseeds and sesame seeds, has been found to reduce breast cancer risk and improve the breast cancer-specific survival of postmenopausal women [34,45,46,47,48,49,50]. Moreover, serum enterolactone levels were positively and significantly correlated with improved prognosis in postmenopausal women with breast cancer [51]. These epidemiological findings suggest the unique suppressive activity of lignans against breast cancer risks in elderly women.

Oral lariciresinol was found to suppress tumor growth and angiogenesis in nude mice implanted with human MCF-7 breast cancer via the induction of apoptosis and the up-regulation of ERβ expression [35]. SDG potently inhibited cell proliferation and induced the apoptosis of breast cancer cells via the down-regulation of ER- and growth factor-mediated gene expression in athymic mice [52]. Sesamin reduced signaling downstream of mitogen-activated protein kinase [53], and is likely to more potently reduce breast tumor growth, compared to SDG [53]. Consistent with the abundance of various lignans in several foods including flax or sesame seeds and oils, these pharmacological effects suggest that lignans are promising dietary compounds for the prevention of breast cancer. PTOX and its structurally related natural compounds exhibit the suppressive activity on mitotic spindle assembly by binding to tubulin, resulting in cell cycle arrest at metaphase [18]. The PTOX semi-synthetic derivatives, etoposide, teniposide, and etopophos (Figure 1B), are clinically utilized to treat certain types of cancers, including testicular/small-cell lung cancer, acute leukemia, Hodgkin’s and non-Hodgkin’s lymphoma [53,54]. These PTOX-derived anti-tumor drugs induce apoptosis of tumor cells by binding to topoisimease II, a key enzyme for cell division [53,54]. In addition, other new PTOX derivatives, including GP-11, NK-611, TOP-53, GL-331, and NPF, are undergoing phase I or II clinical trials as novel cancer drugs [54]. Combined with the difficulty in efficient chemical synthesis of PTOX due to its complicated structure, these findings highlight the importance of PTOX as a natural seed material for the production of various anti-cancer drugs.

In combination, these epidemiological and physiological studies demonstrate that lignans exert diverse, but specific, beneficial effects as dietary compounds or medicinal agents for the prevention of lifestyle-related diseases, such as cancer and diabetes of note, respective lignans exhibit both similar and differential bioactivities in mammals, leading to requirements for the efficient and specific production of these compounds.

## 3. Lignan Biosynthesis Pathways

**Figure 2:**
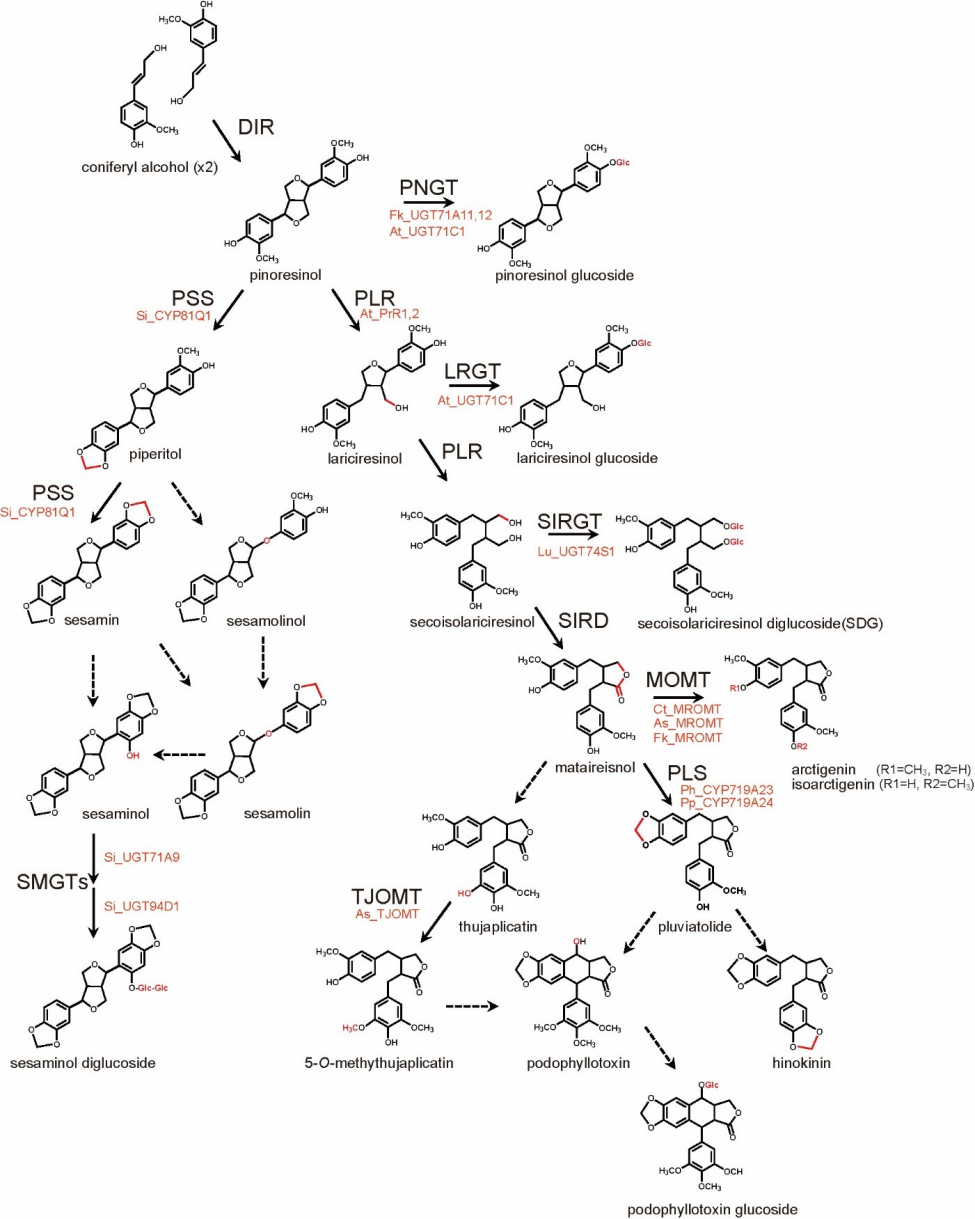
Biosynthesis Pathways of Major Lignans. Chemical Conversions at Each Step are Indicated in Red. Solid and Broken Lines Represent Identified and Unidentified Enzyme-catalyzed Reactions, Respectively.

To date, two major lignan biosynthesis pathways have been identified. Both of the pathways originate from the coupling of achiral *E*-coniferyl alcohol, leading to the generation of pinoresinol, a basal lignan (Figure 2). Although a pinoresinol synthase has yet to be identifed, a dirigent protein (DIR) was shown to participate in the stereo-specific dimerization of *E*-coniferyl alcohol [55]. In diverse plant species including *Forsythia*, *Linum*, and *Podophyllum*, pinoresinol is stepwisely reduced to lariciresinol and then secoisolariciresinol by pinoresinol-lariciresinol reductase (PLR), a member of the pinoresinol-lariciresinol/isoflavone/phenylcoumaran benzylic ether reductase (PIP) family [55,56,57,58,59,60,61]. PLR converts pinoresinol to secoisolariciresinol *via* lariciresinol (Figure 2). Pinoresinol also undergoes glucosylation by UGT71A18, a UDP-glucose-dependent glucosyltranferase [62]. Such glycosylation is highly likely to suppress the chemical reactivity of a phenolic hydroxyl group of pinoresinol and to potentiate high water solubility of pinoresinol aglycone, resulting in large and stable amounts of pinoresinol [1,2,11,12]. Indeed, approximately 90% of pinoresinol is accumulated in its glucosylated form in *Forsythia* spp. [63,64]. PLR-catalyzed metabolism and UGT71A18-directed glucosylation are reciprocally competitive pathways (Figure 2), given that both of them share pinoresinol as a substrate. Intriguingly, *PLR* shows opposite seasonal alteration in gene expression against *UGT71A18*; in *Forsythia* leaves in Japan, *PLR* gene is intensely expressed from April to August but poorly from September to November, whereas gene expression of *UGT71A18* is observed at high level from September to November, but at faint or no level from April to August, at least, in Japan [64]. These findings indicate that PLR and UGT71A18 participate in the competitive regulation of lignan biosynthesis via pinoresinol metabolism. In *A. thaliana*, AtPrR1 and 2 are only responsible for the reduction of pinoresinol to lariciresinol [60], and lariciresinol and pinoresinol are glucosylated by another novel UDP-glucose-dependent glucosyltranferase, UGT71C1 [65].

Secoisolariciresinol, like pinoresinol and lariciresinol, undergoes two metabolic pathways (Figure 2). First, Secoisolariciresinol is converted into matairesinol by secoisolariciresinol dehydrogenase (SIRD) [66]. Second, a novel UDP-glucose-dependent glucosyltranferase in *Linum*, UGT74S1, generates secoisolariciresinol monoglucoside and SDG [67]. Matairesinol is metabolized to arctigenin (Figure 2) by matairesinol *O*-methytransferase (MOMT) via methylation of a phenolic hydroxyl group in various plants including *F. koreana*, *Carthamus tinctorius*, and *Anthriscus sylvestris* [68,69]. Additionally, 70%–90% of matairesinol is glucosylated throughout the year in the *Forsythia* leaves [64], although no matairesinol-glucosylating enzymes have been identified. In *Linum*, *Anthriscus*, and *Podophyllum* plants, matairesinol is also converted into hinokinin, yatein, or PTOX via multiple biosynthetic pathways, although all of the relevant enzymes have not yet been identified [1,2,55]. In *A. sylvestris*, AsTJOMT exclusively methylates the 5-hydroxyl group of thujaplicatin, an intermediate of PTOX [70]. The homologous enzymes, CYP719A23 (from *P.* *hexandrum*) and CYP719A24 (from *P. peltatum*) participate in the conversion of matairesinol into pluviatolide, a more downstream intermediate of PTOX (Figure 2), via methylenedioxy bridge formation [71].

PLR and the downstream biosynthetic enzymes are absent in *Sesamum* plants [1,2,12,55,72,73,74]. Instead, pinoresinol is metabolized into piperitol, followed by further conversion into (+)-sesamin by a cytochrome P450 family enzyme, CYP81Q1, which is responsible for via formation of two methylenedioxy bridges [75]. The *CYP81Q1* gene is expressed almost exclusively in sesame seeds, which is compatible with sesamin production at the highest level in sesame seeds [75]. Sesamin is anticipated to be further metabolized into sesaminol and sesamolin (Figure 2), but the underlying molecular mechanisms have yet to be elucidated [3,55]. Sesaminol is glucosylated at its 2-hydroxyl group by the homologous enzymes UGT71A8 (*S. radiatum*), 9 (*S. indicium*), and 10 (*S. alatum*). Moreover, the resultant sesaminol 2-*O*-monoglucoside is further glucosylated by UGT94D1, which is specific to the glucosylation of sesaminol 2-*O*-monoglucoside at 6-position of the conjugated glucose [76].

A number of key lignan biosynthetic enzymes remain to be identified. Over the past few years, however, the genomes or transcriptomes of lignan-rich plants including *Linum* [77,78,79], *Sesamum* [72,73,74], and *Podophyllum* [71,80,81] have been documented, followed by *in silico* detection of functional genes. Particularly, Next-generation sequencing (NGS) is a promising approach for the molecular characterization of lignan biosynthetic enzymes; indeed, CYP719A23 and its homolog were identified by NGS-based transcriptome [71]. These findings are expected to remarkably enhance the molecular and functional characterization of lignan biosynthetic enzymes. In addition, it is suggested that a *Podophyllum* endophyte may produce PTOX [82]. NGS analyses of the genome, metagenome, and transcriptome of *Podophyllum* and its endophytes are expected to provide crucial clues to understand the PTOX biosynthesis pathways.

## 4. Metabolic Engineering of Lignan Biosynthesis

To date, cell and organ cultures have been employed for metabolic engineering of lignan biosynthesis. Furthermore, a growing body of studies has revealed that lignan biosynthesis is altered by genetic modification, light, and elicitors. This section presents an overview and discussion of recent progress in typical lignan metabolic engineering using plants, plant cells and organ cultures.

### 4.1. Gene Transfection or Silencing

Stable or transient transfection or gene silencing of a lignan biosynthetic enzyme gene is expected to directly alter the lignin production cascades in host plants, organs, and cells, following the development of methods for gene transfection into hosts of interest.

**Figure 3:**
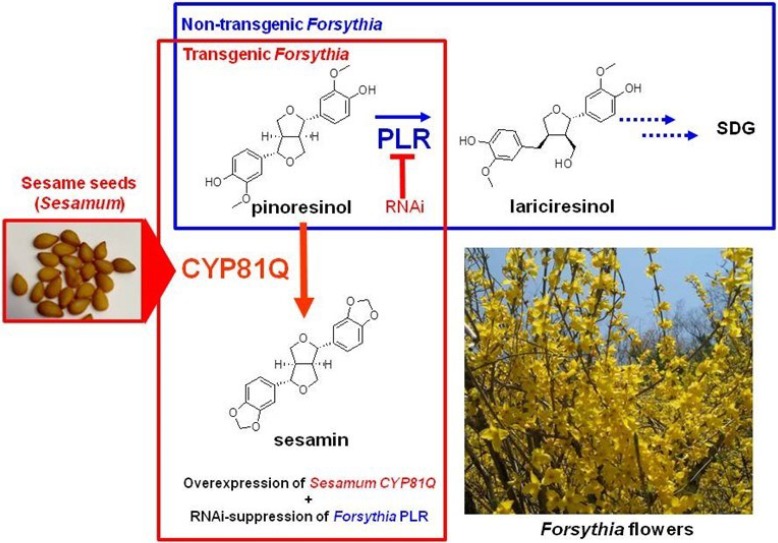
Metabolic Engineering of *Forsythia* Suspension Cell Cultures. The Transgenic *Forsythia* Suspension Cell Culture, CPi-Fk cells, Acquired the Ability to Produce Sesamin by Stable Transfection of *PLR*-RNAi and the *Sesamum* *CYP81Q1* Gene.

*Forsythia* is a perennial plant commonly known as the golden bell flower, and is used for a variety of Chinese medicines and health diets [1,2,5,7,12,55]. As shown in Figure 2, *Forsythia* biosynthesizes pinoresinol, phillygenin, secoisolariciresinol, matairesinol, and arctigenin, with >90% of pinoresinol, > 80% of matairesiol, and 40%–80% of arctigenin accumulated in glucosylated forms [1,2,63,64,83]. Identification of these lignans and the relevant biosynthetic enzymes suggests the potential of *Forsythia* as a platform for lignan production. Although efficient methods for the generation of transgenic *Forsythia* species have not yet been established [84], the metabolic engineering of *Forsythia* culture cells was originally reported. *Forsythia* suspension cells stably transfected with a PLR-RNA interference (RNAi) sequence (*PLR*-RNAi) showed complete loss of matairesinol and an approximately 20-fold increase in total pinoresinol (pinoresinol aglycone and glucoside), compared with the wildtype cells [63]. Furthermore, *Forsythia* transgenic cells, CPi-Fk, which are stably double-transfected with *PLR*-RNAi and the sesamin-producing enzyme, *CYP81Q1* (Figure 2), produced sesamin (0.01 mg/g dry weight of the cell [DW]) (Figure 3), although sesamin is not biosynthesized in native *Forsythia* [63]. This is the first success in lignan metabolic engineering leading to an exogenous lignan using transgenic plant cells. In addition, the RNAi-based suppression of *UGT71A18* (encoding a pinoresinol-glucosylating enzyme) may lead to the dramatic improvement of sesamin production in CPi-Fk cells, given that pinoresinol glucoside cannot be utilized by CYP81Q1 as a substrate [75], and 90% of pinoresinol is glucosylated in *Forsythia* cells [1,2,63,64,83]. Thus, the *Forsythia* cell culture system is an efficient and promising platform for producing both endogenous and exogenous lignans by transgenic metabolic engineering.

RNAi-based metabolic engineering was attempted in various *Linum* species to produce endogenous lignans. Hairy roots of *L. perenne* transiently transfected with *PLR*-RNAi reduced the production of the major endogenous lignan, justicidin B, to 25%, compared with the untreated hairy roots [58]. Likewise, transient transfection of *L. corymbulosum* hairy roots with PLR-RNAi resulted in a marked reduction of hinokinin [59]. Combined with the justicidin B and hinokinin biosynthetic pathways, in which PLR converts pinoresinol into secoisolariciresinol (Figure 2), these findings indicate that PLR-directed conversion of pinoresinol into secoisolariciresinol is a rate-limiting step in justicidin B and hinokinin biosynthesis, at least in the hairy roots of *L. perenne* and *L. corymbulosum,* respectively. Identification and genetic manipulation of justicidin B and hinokinin synthase will contribute a great deal to the establishment of procedures for the direct metabolic engineering of these lignans. Seed coats of *PLR*-RNAi-transgenic plants of *L. usitatissimum* showed the high accumulation of pinoresinol diglucoside and loss of SDG [84]. Intriguingly, these *PLR*-RNAi-transgenic plants produced the 8-5’ linked neolignans, dehydrodiconifnyl alcohol and dihydro- dehydrodiconifnyl alcohol, neither of which was detected in the wildtype plants [84]. Taken together, these findings reinforce the potential of *Forsythia* and *Linum* transgenic or transiently gene-transfected cells and plants as the metabolic engineering-based platforms for on-demand production of both endogenous and exogenous lignans. The draft genome and transcriptome of *Linum* *usitatissimum* [77,78,79] will accelerate the identification of the enzymes involved in the biosynthesis of *Linum* lignans, leading to the efficient lignan production using gene-modified plant sources.

Two factors should be considered in constructing gene-modified plant platforms for lignan production, the type of host and the use of transgenic or transiently transfected hosts. Host types can include plants, organs, and cell cultures. For example, although the amount of sesamin produced by CPi-Fk cells is 100- to 200-fold lower than that by native sesame seeds, CPi-Fk-based lignan metabolic engineering has several advantages. CPi-Fk cells proliferate 10-fold in two weeks in standard culture medium [63], and can be cultivated at all times and locations, whereas sesame seeds are cultivated in limited regions only once a year. Moreover, the conditions used in the culturing CPi-Fk cells, including temperature, light wavelength and intensity, and medium components, can be altered to optimize sesamin production. *Forsythia* plants have much greater biomass, with higher amount of lignans, than suspension cell cultures, and these plants can grow and propagate from small explants without flowering or seed formation. However, efficient generation of transgenic *Forsythia* plants still requires further basic research due to the markedly low transformation efficiency by any known gene transfection methods and deviation among *Forsythia* species [85,86,87]. In contrast, the generation of both stable (namely transgenic) and transient transfectants of *Linum* species are well established, and thus, the amounts of precursors or intermediates of targeted lignans are major determinants for the employment of cell cultures, organ cultures, or plants as host platforms. Additionally, gene-modified host plants may fail to normally grow or to produce lignans of interest due to pytotoxicity of lignans, although the underlying molecular mechanisms have not fully been elucidated [3,11,12,18,19,20,22]. Therefore, generation of lignan-producing plants using multiple plant species is occasionally required.

The second factor involves construction of either transgenic or transiently transfected hosts. Transgenic plants and cell cultures, once generated, are sustainably used for lignan production and readily up-scaled, whereas generation of transgenic plants, in particular non-model plants, may consume time and costs. Moreover, cultivation of transgenic plants in general requires a closed facility for gene-stably modified plants. Transiently transfected plants require repeated transfections, and transient transfection of multiple genes is likely to reduce the transfection efficiency. In addition, massive transient transfection remains to be fully developed [88]. Further research on lignan metabolic engineering, using transgenic or transiently gene-transfected plants, organ cultures, and cell cultures, is expected to lead to the establishment of both universal and molecular species-specific strategies for gene-regulated metabolic engineering of lignan biosynthesis pathways.

### 4.2. Light Irradiation

Biosynthesis of several secondary metabolites, including anthocyanin, carotenoid, and shikonin, is affected by wavelength [89,90,91,92]. Light irradiation has also been shown to improve the production of both endogenous and exogenous lignans by CPi-Fk cells. Irradiation of CPi-Fk cells for two weeks with white fluorescent, blue LED, and red LED light increased sesamin production 2.3-, 2.7-, and 1.6-fold, respectively, compared with cells cultured in the dark [93]. Likewise, irradiation of CPi-Fk cells increased pinoresinol (aglycone and glucoside) production 1.5 to 3.0-fold [93]. Intriguingly, expression of the pinoresinol-glucosylating enzyme UGT71A18 was also downregulated in CPi-Fk cells under blue LED or red LED light, leading to the increase of sesamin production [93], given that pinoresinol glucoside is not metabolized into sesamin by CYP81Q1 [12,75]. In *Linum* species, suspension of *L. album* cells produced two-fold more PTOX under red light than those in the dark [94]. Compared with white fluorescent light, irradiation of *S. indicum* leaves 3–5 weeks after sowing with blue LED light increased sesamin content 2.0-fold, whereas irradiation with red LED light reduced sesamin content two-fold [9,95]. Although the underlying molecular mechanism has yet to be clarified, light irradiation can also improve lignan productivity by both cell cultures and plants.

### 4.3. Elicitation

Plants defense systems are triggered upon injury or infection via signaling by the phytohormones, methyl jasmonate (MeJA) and salicylic acid (SA), and treatment with elicitors, including fungi, their extracts and the glycan components, MeJA and SA, also mimic such activation. Moreover, lignans, at least in part, are believed to be involved in host defense systems [12,18,96]. In combination, elicitors are expected to enhance lignan biosynthesis [18,97]. As summarized in Table 1, the effects of various elicitors on lignan production have been examined in a wide variety of cell cultures and hairy roots of *Forsythia, Juniperus,* and *Podophyllum* (Table 1). MeJA and SA were found to increase the production of PTOX and the structurally related lignan production or the gene expression of lignan biosynthetic enzymes responsible for synthesis of conifenyl alcohol, phenylalanine ammonialyase (PAL), cinnamoyl-CoA reductase (CCR), and cinnammylalcohol dehydrogenasein (CAD) in cell suspension cultures of *L. album* [98,99], and *L. nodiflorum* [98], *Podophyllum hexandrum* [100] and callus of *L. austriacum* callus culture [101]. These phytohormones also increased the PTOX production or the relevant gene expression in hairy roots of *L. tauricum* [102]. Additionally, an increase in production of pinoresinol and matairesinol by MeJA was observed in *Forsythia intermedia* cell suspension culture [103]. Chitosan, chitin oligomers, and other glycans also enhanced PTOX production or gene expression of lignan biosynthetic enzymes in *Juniperus chinensis* callus culture [104], *L. austriacum* callus culture [101], and *L. album* cell suspension culture and hairy roots [105,106,107]. In particular, comparisons of chitin tetramer, pentamer, and hexamer and chitosan tetramer and pentamer showed that treatment of *L. album* hairy roots with chitosan hexamer for five days most potently enhanced PTOX and lariciresinol production, as well as upregulating the expression of *PAL*, *CCR*, *CAD*, and *PLR* genes [107]. Overall, treatment with these elicitors resulted in 2- to 7-fold increases in PTOX synthesis and expression of genes encoding enzymes involved in the early steps of lignan biosynthesis in various plant cells and hairy roots.

Fungal co-culturing, extracts, and filtrate exhibited unique effects on the metabolic engineering of lignan production (Table 1). *Botrytis cinerea*, *Phoma exigua* and *Fusarium oxysporum* extracts triggered the accumulation of monolignols, and enhanced PAL activity and gene expression of *PAL*, *CCR* and *CAD* in *L. usitatissimum* cell suspension cultures [108]. Treatment of in *L. album* cell cultures with *Fusarium graminearum* extract for five days increased PTOX 7.0-fold and PAL, CCR, and CAD mRNAs >10-fold, compared with untreated cells, indicating that this extract is a more potent elicitor of PTOX production and PAL, CCR, and CAD expression than treatment with chitosan, chitin, or MeJA treatment for three days [105,106,109]. In contrast, *Rhizopus stolonifer* and *Rhizoctonia solani* extract stimulated 8.8-fold and 6.7-fold greater accumulation of lariciresinol, instead of PTOX, in *L. album* cell cultures after five-day treatment, as compared with untreated cells, and the highest (6.5-fold) *PLR* gene induction was observed in *L. album* cell cultures treated with *Rhizopus stolonifer* extract for two days [109]. Similar data were obtained in *L. album* hairy roots with the same fungal extracts [106] or *L. album* cell suspension culture with *Fusarium graminearum* culture filtrate [110], but the latter exhibited less lignan production. These studies revealed that fungal extract exhibited species-specific effects on the lignan biosynthesis pathways, although investigation of the molecular basis awaits further study. Examination of the regulation of gene expression has thus far been restricted to enzymes responsible for the upstream of lignan biosynthesis pathways, indicating that the effects of elicitors on the gene expression of the enzymes involved in the downstream of lignan biosynthesis such as SIRD and CYP719A23, and lignan glucosyltransferases (Figure 2) will lead to the identification of more effective elicitors for lignan production.

**Table 1:** List of Major Elicitors and Their Effects on Lignan Biosynthesis.

Elicitor	Target	Effect	References
Chito-oligosaccharides (1 mg)	*Juniperus chinensis* callus culture	Increased PTOX production	[104]
Methyl jasmonate (MeJA) (100 *μ*M)	*Forsythia intermedia* cell suspension culture	Increased pinoresinol and matairesinol production	[103]
Mannan (0.1 mg mL^-1^) β-1,3-glucan (0.1 mg mL^-1^) Ancymidol (10^-7^ M)	*L. austriacum* callus culture	Enhanced activity of tyrosine ammonia-lyase (TAL), coumarate 3-hydroxylase (C3H), polyphenoloxidase (PPO) and PAL	[101]
		Increased PTOX, 6-MPTOX, dPTOX, α- and β-peltatins production	
		Increaded PTOX and α-peltatins production Increaded PTOX, 6-MPTOX, dPTOX and α- peltatins production	
Indanoyl-isoleucine (5-100 µM) Coronalon, (10-50 µM) MeJA(100 *μ*M)	*L. nodiflorum* cell suspension culture	Increased deoxypodophyllotoxin production	[97]
		Enhanced activity of 6-hydroxylase and β -peltatin 6-O-methyltransferas,	
		Increased 6-MPTOX and 5’-d-6-MPTOX production	
MeJA (100 *μ*M)	*L. album* cell suspension culture	Increased PTOX production	[98]
*Botrytis cinerea* extract (3 % v/v)	*L. usitatissimum* cell suspension culture	Rapid stimulation of the monolignol pathway, enhanced PAL activity and expression of genes encoding PAL, CCR and CAD	[108]
*Phoma exigua* extract (3 % v/v)			
*Fusarium oxysporum* extract (3 % v/v)			
MeJA (50–200 μM)	*L. tauricum* hairy root culture	Increased 6MPTOX and 4’-DM­6MPTOX production	[102]
Salicylic acid (SA) (10 μM )	*L. album* cell suspension culture	Enhanced *PAL*, *CCR* and *CAD* gene expression and PTOX production	[99]
Chitin (100 mg l^-1^)	*L. album* cell suspension culture	Increased lariciresinol and/or PTOX production	[105]
Chitosan (100–200 mg L^-1^)			
MeJA (100–200 *μ*M)			
*Fusarium graminearum* extract (1 % v/v)	*L. album* cell suspension culture	Enhanced *PAL*, *CCR*, *CAD*, and *PLR* gene expression Increased PTOX and lariciresinol production	[105,109]
*Sclerotinia sclerotiorum* extract (1 % v/v)			
*Rhizopus stolonifer* extract (1 % v/v)			
*Rhizoctonia solani* extract (1 % v/v)			
MeJA (10–100 μM)	*Podophyllum hexandrum* cell suspension cultute	Changes in cell proteome, Increased PTOX production	[109]
*Fusarium graminearum* extract (1 %v/v)	*L. album* hairy root culture	Enhanced *PAL*, *CCR*, *CAD* and *PLR* gene expression, Increased PTOX, 6MPTOX, and lariciresinol production	[106]
*Sclerotinia sclerotiorum* extract (1 %v/v)			
*Trichoderma viride* extract (1 %v/v)			
Chitosan (100 mg l^-1^)			
Chitosan and chitin oligomers (100 mg L^-1^)	*L. album* cell suspension culture	Enhanced *PAL*, *CCR*, *CAD* and *PLR* gene expression,	[107]
		Increased PTOX, 6MPTOX . and lariciresinol production	
*Fusarium graminearum* culture filtrate (1 % v/v)	*L. album* cell suspension culture	Increased phenolic compound, PTOX and lariciresinol production	[110]
		Enhanced PAL activity,	

## 5. Conclusions

There have been many recent advances in metabolic engineering for lignan production by plants, including: (i) the molecular characterization of novel genes encoding enzymes for biosynthesis pathways of dietary and medicinal lignans; (ii) the production of both endogenous and exogenous lignans by transient or stable transfection of lignan biosynthetic genes into cultured cells, tissues and plants; and (iii) the identification of exogenous stimuli such as light and elicitors that increase the production of lignans by cultured cells and plants. Taken together, combination of gene transfection, light, and elicitors is a promising strategy for further improvement of the lignan productivity; *e.g.*, elicitation of CPi-Fk under blue or red LED light is expected to increase the amounts of sesamin. Moreover, bioinformatic analysis based on the aforementioned experimental data will lead to the systematic prediction of optimal lignan production strategy: hosts (cells, organ cultures, plants), light conditions, elicitor types, and transfection types, given that different optimal production of respective lignans is highly likely to occur under different conditions. For example, three *Forsythia* varieties, *F. koreana*, *F. intermedia,* and *F. suspensa* displayed differential growth and regeneration in a medium component- or selection marker antibiotics-dependent fashions [84], and *Linum* spp. showed genus-specific sensitivities to different elicitors (Table 1).

Public acceptance of dietary products derived from transgenic organisms is limited. Nevertheless, lignans produced by transgenic hosts are chemically identical to natural ones, and free from any recombinant genes or proteins. Thus, their public acceptance is expected to be more easily garnered than that of transgenic foods. Accordingly, more attention should be paid to the establishment of scaling-up and following industrialization of the lignan production systems [111,112,113]. Large-scale lignan production by transgenic plants requires a closed cultivation system to prevent contamination of the environment by transgenic plants. Recently, various closed plant factories have been emerging, which completely shut off a gene flow into the outer environment and enables the transgenic plants-based molecular breeding of genes or compounds of interest under optimal and sterile conditions [111,112,113]. Such advances in the metabolic engineering of lignan biosynthesis, combined with the aforementioned outcomes of a wide range of basic research, will surely pave the way for the conversion of conventional agricultural lignan production to innovative industrial lignan production.
